# Adequate hemodialysis improves anemia by enhancing glucose-6-phosphate dehydrogenase activity in patients with end-stage renal disease

**DOI:** 10.1186/1471-2369-15-155

**Published:** 2014-09-26

**Authors:** Mahmoud Husni Ayesh (Haj Yousef), Ahnaf Bataineh, Elham Elamin, Yousef Khader, Khaldoon Alawneh, Mohamad Rababah

**Affiliations:** Department of Internal Medicine, King Abdullah University Hospital, Faculty of Medicine, Jordan University of Science and Technology, P.O. Box 3030, Irbid, 22110 Jordan; Saad specialist hospital, Al Khobar, Saudi Arabia; Faculty of Medicine & Health Sciences, El Imam Elmahdi University, P.O. Box 209, Khartoum, 11588 Sudan; Department of Public Health, Faculty of Medicine, Jordan University of Science and Technology, P.O. Box 3030, Irbid, 22110 Jordan

**Keywords:** Anemia, Glucose-6-phosphate dehydrogenase, Hemodialysis

## Abstract

**Background:**

We conducted this study to determine the erythrocyte glucose-6-phosphate dehydrogenase (G6PD) activity level in patients with end-stage renal disease (ESRD) on maintenance hemodialysis (HD) and to determine the effect of hemodialysis adequacy on G6PD activity levels and its impact on anemia.

**Methods:**

Eighty-two patients (48 men and 34 women) receiving regular hemodialysis for ESRD through arteriovenous fistulae for at least one year prior to the start of the study were enrolled in this study. G6PD activity levels were measured in all patients and the average Kt/V was used as a parameter of HD adequacy. Patients were divided into two groups according to Kt/V values. Group 1 included 45 patients with Kt/V˃1.2 (adequate HD), and group 2 included 37 patients with Kt/V˃1.2 (inadequate HD). The average hemoglobin level and the weekly dose of an erythropoietin-stimulating agent, epoetin alpha (ESA), for each patient were calculated for one year.

**Results:**

The mean (SD) erythrocyte G6PD activity for all patients on hemodialysis was 7.64 ± 1.85 U/g Hb. Patients who had received adequate hemodialysis had a significantly higher average erythrocyte G6PD (mean (SD) = 9.2 ± 0.7 U/g Hb) compared to patients who had inadequate hemodialysis (mean (SD) = 5.7 ± 0.7U/g Hb) (*P*-value <0.005). The mean hemoglobin concentration was significantly higher in patients with adequate hemodialysis compared to those with inadequate hemodialysis.

**Conclusion:**

Our study demonstrated the beneficial effect of adequate hemodialysis in correcting anemia by enhancing the erythrocyte G6PD activity in patients.

## Background

Factors contributing to anemia in ESRD patients include erythropoietin deficiency, blood loss, shortened red blood cell (RBC) life span, vitamin deficiencies, iron deficiency, and chronic inflammation [1, 2]. The shortened RBC survival observed in uremic patients has been attributed mainly to uremic toxins [3]. In patients with ESRD, there is growing evidence that increased oxidative stress may contribute to anemia, along with other factors such as erythropoietin-stimulating agent (ESA) resistance, malnutrition, and atherosclerosis [4].

Erythrocyte G6PD is the key enzyme in the hexose monophosphate shunt (HMP). The HMP plays a significant role in RBC antioxidant reactions and is the only source of nicotinamide adenine dinucleotide phosphate (NADPH), a molecule that is essential in maintaining reduced glutathione (GSH) levels. In vitro studies have shown that low-molecule uremic toxins methylguanidine (MG) and guanidinosuccinic acid (GSA) have negative effects on the activity of erythrocyte G6PD in human RBCs [5]. Several studies have shown decreased G6PD activity in uremic patients. This decrease in erythrocyte G6PD activity causes hemolysis, and likely plays a role in the pathogenesis of anemia in patients with ESRD [4, 6].

Hemodialysis, per se, and the adequacy of hemodialysis measured by Kt/V have been shown to play an important role in improving anemia and reducing the ESA dosage required for anemia correction in patients with ESRD [7–11]. This benefit may due to the correction of oxidative stress, and the removal of molecules that inhibit erythropoiesis and erythrocyte G6PD activity [12]. This effect may explain the beneficial effect of hemodialysis adequacy, using Kt/Vas a parameter for the assessment of dialysis adequacy, on anemia in patients with ESRD on maintenance HD. However, there are no data available regarding the effect of hemodialysis adequacy on the activity of erythrocyte G6PD.

The Kt/V ratio is one parameter used for measurement of the adequacy of dialysis treatment. In this expression K is dialyzer clearance, expressed in mL/min, t indicates the duration of the dialysis session, and V is the volume of water the patient’s body. Clearance multiplied by time (Kt) is a measure of the volume of fluid completely cleared of urea during a single dialysis treatment.

We conducted this study to determine the G6PD activity level in patients with ESRD on maintenance HD and to study the effect of hemodialysis adequacy on G6PD activity levels and its impact on anemia.

## Methods

### Patients

This study was performed over a period of one year in the outpatient dialysis unit of King Abdullah University Hospital, an 800-bed tertiary care center. Eighty-two patients (48 men and 34 women) receiving regular hemodialysis for ESRD through arteriovenous fistulae for at least one year prior to the start of the study were enrolled in this study. Information about the underlying cause of ESRD and Kt/V average over a period of one year were obtained from their dialysis unit records. The underlying causes of ESRD were divided into two categories: diabetic nephropathy in 39 patients, and non-diabetic nephropathy 43 patients. Patients underwent dialysis three times weekly with sessions consistently four hours in duration. At the time of enrollment, complete blood count with red cell indices, reticulocyte percentage and count, liver function tests, kidney function tests, serum ferritin, serum iron, serum vitamin B12, and serum folate levels were measured in all patients. Direct antiglobulin test, parathyroid hormone level, and C-reactive protein (CRP) were also measured. Patients were also screened for hepatitis B and C. Patients were grouped in to 2 groups according to their Kt/V. Group 1 included 45 patients with Kt/V˃1.2 (adequate HD) and group 2 included 37 patients with Kt/V˃1.2 (inadequate HD). All patients underwent ultrasound (US) examination of the kidneys to evaluate for cystic lesions.

The Human Research Committee at Jordan University of Science and Technology and the Institutional Review Board of King Abdullah University approved this study. All patients signed written informed consent prior to their participation.

### Inclusion and exclusion criteria

Patients with ESRD on regular maintenance hemodialysis for at least one year prior to the start of the study, were included in this study. All patients were iron replete and had received ESA for at least four months prior to the start of the study. Patients were excluded from this study if they had received a blood transfusion in the four months prior to the start of the study. Patients with congenital polycystic kidney diseases and acquired cystic kidney diseases detected by US imaging were excluded. Patients with any known blood disorders including G6PD deficiency, hemoglobinopathy, elevated white blood cell count >12 × 10^9^/L and platelet count >600 × 10^9^/L were excluded. Patients with anemia not due to ESRD including iron deficiency, vitamin B12 deficiency, and folate deficiency were also excluded. Patients on maintenance HD with evidence of inflammation (as suggested by high CRP levels) were excluded from this study. Additionally, patients with hepatic disease, respiratory disease, and those taking antioxidant vitamin and fish oil supplements were excluded from this study.

### ESA

All patients with ESRD on maintenance HD included in the study were on the same type ESA administered on a per kilogram basis. The average weekly dose over the course of one year was calculated for each patient.

### Erythrocyte G6PD activity level

Six ml of venous blood was collected from each patient just before the HD session to measure average erythrocyte G6PD activity. Quantitative assay of erythrocyte G6PD level was performed on freshly prepared hemolysates using a commercial kit (Trinity Biotech USA). This method employs a modification of the spectrophotometric method. The assay of erythrocytes G6PD activity was begun with a determination of the hemoglobin level (Hb g/dl) in an ethylenediaminetetraacetic acid (EDTA) blood sample. The hemolysate samples were subjected to RBC enzyme analysis as described by Beutler et al. [13]. G6PD activity in the hemolysate was measured by spectrophotometric assay (Beckman Coulter, CX9 USA) at 340 nm and at 37C within 0–19 min after the hemolysate was introduced into the CX9 system. The calculated G6PD activity U/L was converted to G6PD activity U/G Hb as follow:


The normal value was established by measuring erythrocyte G6PD activity in the control group. The normal value for erythrocyte G6PD was considered as the mean of control ± SD.

### Kt/V and dialysis modality

Standard HD had been provided for all patients using HD instruments (Fresenius 4000H machines) with online measurement capabilities. All patients used synthetic Polysulfone F series high performance steam capillary dialysis designed for single use (Fresenius medical, USA). Patients were considered to have adequate hemodialysis if their Kt/V ratio was >1.2 [14].

### Statistical analysis

Statistical analysis was performed using the Statistical Package for Social Science (SPSS) software. The data were described using means and standard deviations. Two-tailed *t* test was used to determine the statistical significance of the differences between two means. Fisher Exact test was used to compare the difference between two proportions. A *P-*value of less than 0.05 was considered statistically significant.

## Results

### Patients’ characteristics

This study included 82 patients [48 male (58.5%) and 34 female (41.5%)] with a mean (SD) age of 50.3 ± 20.7 years. The demographic and clinical characteristics of patients shown in Table 1. All patients were treated with the same type ESA (epoetin alpha) according to body weight.

**Table 1:** **The demographic and clinical characteristics of 82 patients receiving regular hemodialysis for ESRD**

Characteristics	Mean ± SD
Sex, n (%)	
Males	48 (58.5)
Females	34 (41.4)
Age (year)	50.32 ± 20.73
Diabetes mellitus, n (%)	
Yes	39 (47.6)
No	43 (52.4)
Hemoglobin (Hb) before initiation of HD (g/dL)	9.33 ± 1.05
Hemoglobin at end of study (g/dL)	11.78 ± 2.05
G6PD activity level at end of study (U/g Hb)	7.64 ± 1.85
Serum creatinine level (μmol/L)	758.45 ± 280.3

### G6PD level and adequacy of dialysis

Table 2 shows the laboratory and clinical characteristics of the patients based on the adequacy of hemodialysis as measured by Kt/V. The mean (SD) erythrocyte G6PD activity for all patients on hemodialysis was 7.64 ± 1.85 U/g Hb. Patients who had received adequate hemodialysis had a significantly higher average erythrocyte G6PD (mean (SD) = 9.2 ± 0.7 U/g Hb) compared to patients who had inadequate hemodialysis (mean (SD) = 5.7 ± 0.7U/g Hb) (*P*-value <0.005). There were no significant differences in the prevalence of diabetes between patients with adequate hemodialysis and those with inadequate hemodialysis. The mean hemoglobin concentration was significantly higher in patients with adequate hemodialysis compared to those with inadequate hemodialysis. The mean average ESA dose was lower in patients with adequate HD compared with those with inadequate HD.

**Table 2:** **The laboratory and clinical characteristics of the patients based on the adequacy of hemodialysis as measured by Kt/V**

Variable	Adequate hemodialysis N = 45	Inadequate hemodialysis N = 37	***P***-value
Kt/V	1.31 ± 0.96	0.98 ± 0.09	0.046
Age, years	49.8 ± 20.6	57.9 ± 16.6	0.057
ESA (epoetin alpha) (IU/Week)	7176.4 ± 2823.6	9155.0 ± 2844.3	0.002
Serum creatinine (μmol/L)	778.0 ± 274.1	776.3 ± 277.0	0.978
G6PD activity level (U/g Hb)	9.2 ± 0.7	5.7 ± 0.7	<0.005
Diabetic patients, n (%)	21 (46.7%)	18 (48.7%)	0.965
Hematocrit	37.0 ± 6.0	28.4 ± 7.2	<0.005
Hemoglobin (g/dL)	12.0 ± 2.0	9.5 ± 2.4	<0.005
Reticulocyte count, %	1.9 ± 0.10	2.2 ± 0.3	<0.005
HD duration (months)	18,06 ± 14.66	15 ± 14.28	0.486

## Discussion

This study showed that there was significant difference in the erythrocyte G6PD activity level in patients with adequate HD (Kt/V ≥1.2) compared to those with inadequate HD (Kt/V ˃1.2). Patients with adequate HD had significantly higher erythrocyte G6PD activity and hemoglobin levels compared to patients who received inadequate HD. Despite prior studies showing lower G6PD activity in diabetes mellitus [15–17], the prevalence of diabetes was not significantly different between our two groups. Therefore, diabetes was unlikely to be a confounding factor in the association between adequate hemodialysis and G6PD activity levels. Interestingly, patients with adequate HD required a lower average weekly dose of ESA to reach the target hemoglobin level over the one year of the study than those with inadequate HD. This supports the theory that hemodialysis adequacy is the main factor responsible for higher G6PD activity levels in these patients. This is in agreement with other studies that have demonstrated that patients with adequate HD had a better response to ESA than those patients with inadequate HD [8, 10].

Adequate HD in our study has been shown to be associated with higher hemoglobin levels in patients on maintenance HD than patients with inadequate HD, although patients with inadequate HD had a higher reticulocyte percentage compared to patients with adequate HD. Despite the fact that reticulocytes had higher G6PD activity than older RBCs, patients with inadequate HD still had lower G6PD activity levels. Moreover, one need to consider that the increased reticulocyte activity in the low Kt/V group compared to the high Kt/V group might indicate adequate response to ESA. Then the decreased Hb concentration in low Kt/V group could be explained by decreased RBC lifespan.

The reduced activity of G6PD might be explained by the high level of oxidized glutathione (GSSG) which is found to be increased in the plasma of patients with chronic renal failure compared to those with normal renal function. Costagliola et al. [18] reported that high levels of GSSG in the plasma could exert important effects on RBCs including inhibition of G6PD activity. With the subsequent alteration of the glutathione system, GSSG readily reacts with hemoglobin to produce hemoglobin-glutathione mixed disulfides, with consequent protein aggregation and precipitation. Several studies have shown improvement in hemoglobin levels in patients with maintenance HD treated by glutathione reduction, this supports our finding of decreased G6PD activity in patients with ESRD on maintenance HD [19].

In our study, we used the same dialysis membrane during HD in all patients in order to separate the effect of HD adequacy on G6PD activity from a potential membrane effect on G6PD activity [20]. In addition, in our study the difference in mean age between the two groups is marginally significant (*P*-value = .057), and this might have contributed to the observed difference in G6PD activity levels. Aging has been associated with a decrease in the G6PD activity level [21], and the inadequate HD group had a higher mean age (57.9 ± 16.6 years) compared to the adequate HD group (49.8 ± 20.6 years).

Our study demonstrates that hemodialysis adequacy plays an important role in correcting anemia in patients with ESRD on regular HD by enhancing the activity of erythrocyte G6PD activity. Hemodialysis per se, without administration of ESA, improves anemia, this suggest that hemodialysis exerts its effect by removal of inhibitors of erythropoiesis [7, 22]. In our study, we suggest that hemodialysis, by removal of G6PD enzyme inhibitors, plays an important role in the improvement of anemia in patients with ESRD as suggested by previous studies [4, 6, 12].

One limitation of this study is that erythrocyte G6PD activity level was not measured at the time of ESRD diagnosis and prior to the institution of HD. Dialyzer membranes have been shown to have antioxidant effects, and may have had an effect on our results. Another limitation of this study is that no patients with inadequate HD had their G6PD activity measured after increasing the dialysis intensity to an adequate HD level (Kt/V ˃1.2). This was not feasible as patients on regular HD have a fixed time and duration for their dialysis session, therefore the reasons for dialysis inadequacy are difficult to correct. Moreover, other markers of intravascular hemolysis such as haptoglobin or LDH were not available for analysis.

## Conclusion

Our study demonstrated the beneficial effect of adequate hemodialysis in correcting anemia by enhancing the erythrocyte G6PD activity in patients. Improving anemia in patients with ESRD may result in reduction of ESA requirements, and may improve the general status of ESRD patients by reducing the morbidity associated with anemia. Additionally, enhancing the activity of a major antioxidant enzyme, such as G6PD, may result in a reduction of complications secondary to increased oxidative stress in patients with ESRD.
